# Curcumol Inhibits the Development of Prostate Cancer by miR-125a/STAT3 Axis

**DOI:** 10.1155/2022/9317402

**Published:** 2022-07-30

**Authors:** Wen Sheng, Jin Ding, Lumei Liu, Neng Wang, Baowei Lu, Xujun You, Qinghu He, Qing Zhou

**Affiliations:** ^1^Andrology Laboratory, Hunan University of Chinese Medicine, Changsha 410208, China; ^2^Department of Andrology, Affiliated Bao'an Hospital of Traditional Chinese Medicine, The Seventh Clinical Medical College, Guangzhou University of Traditional Chinese Medicine, Shenzhen 518133, China; ^3^Andrology Laboratory, Hunan University of Medicine, Huaihua 418000, China; ^4^Department of Andrology, The First Hospital of Hunan University of Chinese Medicine, Changsha 410021, China

## Abstract

**Aim:**

This study aimed to learn the antineoplastic activity of curcumol (Cur) on prostate cancer (PCa) and elucidate its potential molecular mechanism.

**Methods:**

The proliferation, invasion, and migration of PCa cells (PC3 and 22RV1) were detected by the cell counting kit 8 (CCK8), transwell, and wound healing assay, respectively. The expression of genes and proteins was analyzed by quantitative real-time polymerase chain reaction (qRT-PCR) and western blotting (WB), respectively. The protein expression in tissues and cells was tested through immunohistochemistry (IHC) and immunocytochemistry (ICC). Enzyme-linked immunosorbent assay (ELISA) was utilized to quantify the level of epidermal growth factor (EGF) and vascular endothelial growth factor (VEGF). The interaction between microRNA125a (miR-125a) and the signal transducer and activator of transcription 3 (STAT3) was confirmed via dual-luciferase reporter assay.

**Results:**

Cur effectively restrained the proliferation, invasion, and migration of PC3 and 22RV1 cells. After Cur intervention, miR-125a, miR-375, miR-149, miR-183, and miR-106b were all upregulated in PC3 cells, among which miR-125a was the most significantly upregulated. Dual-luciferase reporter assay combined with qRT-PCR and WB experiments confirmed that miR-125a targeted STAT3. Both *in vitro* and *in vivo*, Cur enhanced miR-125a expression and suppressed the activation of the STAT3 pathway in PCa. Also, Cur effectively inhibited the growth of PCa.

**Conclusion:**

Cur inhibited the development of PCa by miR-125a/STAT3 axis. This may provide a potential agent for treating PCa.

## 1. Introduction

Prostate cancer (PCa) is a type of cancer that is observed in men worldwide and is considered an important cause of death in affected males [1]. Mostly, PCa is diagnosed as low-grade cancers with low mortality [2]. At present, PCa is mainly treated by surgery or drugs [3]. Therefore, it is particularly important to develop effective and low toxicity anti-PCa drugs.

Curcumol (Cur), a sesquiterpenoid with pharmacological activity, has multitarget anticancer activities and shows great therapeutic effects against various types of cancer, such as gastric cancer, cholangiocarcinoma, and breast cancer [4–7]. Of note, Ning et al. found that Cur inhibited the proliferation and metastasis of melanoma via the microRNA152-3p (miR-152-3p)/phosphoinositide 3-kinase (PI3K)/serine-threonine kinase (AKT) and the extracellular signal‐regulated kinase (ERK)/nuclear factor-kappa B (NF-*κ*B) signaling pathways [8]. Cur inhibited the Krüppel-like factor 5 (KLF5)-dependent angiogenesis by blocking the reactive oxygen species (ROS)/ERK signaling pathway in the liver sinusoidal endothelial cells [9]. Although there has been evidence that Cur has anti-PCa properties [10], the specific regulatory mechanism of Cur on PCa still needs to be further explored.

miRNAs can guide the posttranscriptional regulation of gene expression through multiple mechanisms [11]. Previous research has found that miR-125a played an important role in regulating the homeostasis and growth of immune cells, as well as cancer cells [12]. For example, miR-125a-5p targeted FUT4 to inhibit the development of bladder cancer [13]. Prior researches have attested that miR-125a was strongly associated with PCa [14]. We have previously found that Cur could inhibit the development of PCa [15]. In this research, we attempted to investigate whether the anti- PCa effect of Cur was related to miR-125a.

In the signal transducer and activator of transcription 3 (STAT3) protein family, STAT3 is a key signaling protein that is activated by diversiform growth factors and cytokines and has gained increasing attention due to its close association with malignant tumors [16]. Generally, STAT3 is regarded as the main regulator and hub of many signal transduction pathways [17]. Functionally, STAT3 is considered to play a carcinogenic role in multiple malignant tumors. In PCa, targeting STAT3 enhances immunogenic cell death induced by oncolytic Newcastle disease virus [18]. Therefore, blocking the abnormal activity of STAT3 in tumor cells may potentially be used as a novel approach to intervene and treat tumors [19]. Interestingly, Cur mediated the interaction of hypoxia-inducible factor (HIF)-1*α* and STAT3 pathways to inhibit the activation of programmed cell death ligand 1 (PD-L1) in hepatic cancer [20]. However, whether Cur has similar interaction with STAT3 in PCa requires further investigation.

The purpose of this research was to probe the effect of Cur on the proliferation, invasion, and migration of PCa cells. We screened out the miRNAs significantly regulated by Cur in PCa cells via bioinformatics analysis and experimental verification and further explored the mechanism of Cur on PCa.

## 2. Materials and Methods

### 2.1. Cell Culture and Treatment

PCa cell lines (PC3, DU145, LNCaP, and 22RV1) and normal prostate cells (RWPE-1) were purchased from Shanghai Zhongqiao Xinzhou Biotechnology Co., Ltd. The culture conditions of PC3, DU145, LNCaP, and RWPE-1 cells were consistent with previous reports [15]. 22RV1 cells were cultured in RPMI-1640 supplemented with 10% fetal bovine serum (FBS) and 1% penicillin/streptomycin. Cur (0, 25, 50, and 100 *μ*g/ml) was used to treat RWPE-1 and PCa cells for 48 h.

### 2.2. Cell Transfection

The chemically synthesized miR-125a mimic, miR-125a inhibitor, small interfering RNA (siRNA) of STAT3, STAT3 overexpression vector, and related negative controls (NC) were purchased from RiboBio (Guangzhou, China). Lipofectamine 2000 (Invitrogen, USA) was applied for transfection.

### 2.3. Cell Counting Kit-8 (CCK8) Assay

Cells were treated with 0, 25, 50, and 100 *μ*g/mL Cur (B20342, Shanghai YuanYe Biotechnology Co., Ltd.) for 48 h. Then, the cells were digested, counted, and added into 96-well plates (1 × 10^4^ cells/well). Every group contained three compound holes. After the corresponding time, CCK8 (Dojindo, Japan) was inoculated to each well (10 *μ*L/well). After incubation with 5% CO_2_ at 37°C for 4 h, the optical density (OD) value was analyzed at 450 nm by a BioTek microplate analyzer.

### 2.4. Transwell Assay

To assess the invasion ability of PCa cells, we took the logarithmically growing cells and placed them in a 6-well plate. Precooled serum-free MEM medium was used to dilute the Matrigel (Biosciences, USA) (Matrigel : medium (v/v) = 1 : 2) to prepare the transwell chambers. Five hundred microliters' complete medium (containing 10% FBS) was added to the lower chambers. Cells were digested with trypsin to form a single cell and resuspended to 2 × 10^6^ cells/mL. 100 *μ*L cells (approximately 2 × 10^5^ cells) were injected to each hole, at 37°C for 48 h. After being fixed with 4% paraformaldehyde for 20 min, the membrane was dyed with 0.1% crystal violet for 5 min. The film was placed on a slide and photographed under a microscope. Next, 500 *μ*L of 10% acetic acid was added to the chamber for decolorization, and the OD value was detected using a BioTek microplate analyzer at 550 nm. Cell migration ability was detected without incubating the cells with Matrigel, and the other steps were the same as above.

### 2.5. Wound Healing Assay

Cells were digested with trypsin. After counting, approximately 5 × 10^5^ cells were added to each well, with a straight line drawn with a marker on the back. After the plate was covered with cells, we scratched the cells with a 10 *μ*L pipette tip perpendicular to the previous straight line. We took a 0 h scratch and captured three visual fields at each time point. After incubation with 5% CO_2_ at 37°C for 24 and 48 h, photos were taken again.

### 2.6. Quantitative Real-Time Polymerase Chain Reaction (qRT-PCR)

TRIzol reagent (Thermo, USA) was performed to extract total RNA from the tissues and cells in each group. RNA was quantified by spectrophotometry, and its quality was assessed by 1.5% agarose gel electrophoresis (AGE) and ethidium bromide (EtBr) dyeing. In accordance with the manufacturer's instructions, the reverse transcripts were synthesized using a Thermo Scientific RevertAid First Strand cDNA Synthesis Kit. Using the SYBR Green Gamma Real-Time PCR detection system (Thermo, USA), specific primers were used to assess the gene transcription levels. U6 and *β*-actin were used as internal parameters, respectively. 2^−ΔΔCt^ method was used to quantitatively analyze the relative expression levels of genes. The primer sequences are listed in Table 1.

### 2.7. Bioinformatics Analysis and Dual-Luciferase Reporter Assay

We downloaded the original datasets of GSE46738 and GSE45604 from the Gene Expression Omnibus (GEO) database. The *R* language limma package was used to process the expression matrix and perform an expression difference analysis. According to the *P* and logFC values, the upregulated and downregulated differentially expressed miRNAs (DE-miRNAs) with statistical significance (*P* < 0.05, |logFC|>0.5) were screened. The prediction of miRNA target genes was analyzed via TargetScan (http://www.targetscan.org/vert_71/), Human microRNA Disease Database (HMDD) (http://www.cuilab.cn/hmdd), miRDB (http://mirdb.org/), miRWalk (http://mirwalk.umm.uni-heidelberg.de/) databases, and DIANA TOOLS (http://diana.imis.athena-innovation.gr/DianaTools/index.php?r=site/index). We predicted the putative binding site between miR-125a-5p and STAT3 using the miRcode website (http://www.mircode.org). The reporter vector consisting of the luciferase gene and subsequent miR-125a binding common sequence was obtained from Signosis, Inc. (Sunnyvale, CA, USA). STAT3 wild-type (wt) or STAT3 mutant (mut) plasmid DNA was transfected into cells. After 24 h, luciferase activity was determined via a dual-luciferase reporter gene assay system (Promega Corporation, Madison, Wisconsin, USA).

### 2.8. Enzyme-Linked Immunosorbent Assay (ELISA)

The levels of epidermal growth factor (EGF) and vascular endothelial growth factor (VEGF) were quantified using specific ELISA kits, following the manufacturer's protocol. EGF and VEGF kits were purchased from Huamei Bioengineering Co., Ltd. (Wuhan, China).

### 2.9. Western Blotting (WB)

The total protein of the sample was acquired after the tissue or cells were lysed with the radio immune precipitation assay (RIPA) buffer (Beyotime, Nanjing, China). As the manufacturer's instructions, protein was quantified by a Bicinchoninic Acid Assay (BCA) protein quantification kit (AWB0104a, Abiowell, China). 100 *μ*L protein supernatant was mixed with 25 *μ*L 5 × loading buffer and denatured by boiling for 5 min. The denatured protein was transferred to NC membrane for sealing. The membrane was incubated with the primary antibody at 4°C overnight. The primary antibodies were listed as follows: anti-STAT3 (1 : 1500, ab68153, Abcam, UK), anti-p-STAT3 (1 : 5000, ab76315, Abcam, UK), and anti-*β*-actin (1 : 5000, 60008-1-Ig, Proteintech, USA). Horseradish peroxidase (HRP) goat anti-mouse IgG (1 : 5000, SA00001-1, Proteintech, USA) or HRP goat anti-rabbit IgG (1 : 6000, SA00001-2, Proteintech, USA) antibody was then incubated with the membrane for 90 min, at room temperature. The ECL chemiluminescence solution was incubated with the membrane for 1 min. The membrane was wrapped with plastic film. Then, the membrane was exposed and developed with *X* film in a dark box or with a Gel Imaging System (Bio-Rad, USA).

### 2.10. *In vivo* Tumorigenesis

Thirty SPF-grade, 6-week-old BALB/c nude mice were randomized into 5 groups (6 mice per group): control, Cur, Cur + miR-125a inhibitor, Cur + miR-125a inhibitor + si-STAT3, and Cur + antagomir-125a + oe-STAT3 groups. The PCa model was established as previously described [21, 22]. Briefly, PC3 cells were transfected with miR-125a inhibitor and si-STAT3 or oe-STAT3 for 24 h. PC3 cells (5 × 10^6^/100 *μ*L in 50% PBS+ 50% Matrigel) were subcutaneously injected into the right side of 6-week-old BALB/c nude mice (Cyagen, Jiangsu). Cur (20 mg/kg) was administered intragastrically every two days. Tumor volume was surveyed (length × width ^2^ × 0.5) twice a week. After 28 days, the mice were euthanized. The tumors were weighed. All of the experiments were conducted in accordance with the recommendation and approval of the Animal Welfare Committee of Hunan University of Chinese Medicine (Approval No. 2019-0019).

### 2.11. Immunocytochemistry (ICC)

The cells fixed by 4% paraformaldehyde were permeated in 0.3% Triton for 30 min and sealed in PBS buffer for 9 min. After inactivation in 3% hydrogen peroxide (H_2_O_2_), the cells were incubated with the Rabbit anti-STAT3 antibody (ab68153, 1 : 100, Abcam, UK) at 4°C overnight. Further, the culture was incubated with rabbit anti-IgG secondary antibody (AP182C, 1 : 400, Millipore, USA) for 30 min. They were then incubated in diaminobenzidine (DAB) working solution for 1–5 min for color development. The cell slides were stained with hematoxylin for 5–10 min and dehydrated. Finally, the cells were observed under a microscope.

### 2.12. Immunohistochemistry (IHC)

Tumor tissue (5 *μ*m) sections were immersed in xylene for dewaxing and then rinsed with graded alcohol for rehydration. After boiling, the pressure cooker antigen was repaired using ethylenediaminetetraacetic acid (EDTA) for 2 min. After inactivation in 3% H_2_O_2_, nonspecific dyeing of the slide was sealed with 3% bovine serum albumin (BSA) for an hour. Tissue sections were incubated with mouse anti-CD31 (ab24590, 1 : 100, Abcam, UK) and rabbit anti-VEGF (19003-1-AP, 1 : 100, PTG, USA) antibodies at 4°C overnight. The HRP polymer anti-mouse/rabbit IHC Kit (Maxvision, Fuzhou, China) was incubated with sections at room temperature for an hour. DAB + chromogen (Maxvision, Fuzhou, China) was prepared for color development. Image-Pro Plus v.5.1 software (Media Cybernetics, USA) was utilized to determine the positive staining rate of each antigen.

### 2.13. Statistical Analysis

Statistical software GraphPad 8.0 was performed for data analysis. The measurement data were described as the mean ± standard deviation (SD). Student's *t*-test and one-way ANOVA were used for data comparison between two or multiple groups. When *P* < 0.05, differences were considered statistically significant.

## 3. Results

### 3.1. Effects of Cur Treatment on Biological Processes of PC3 and 22RV1 Cells

**Figure 1:**
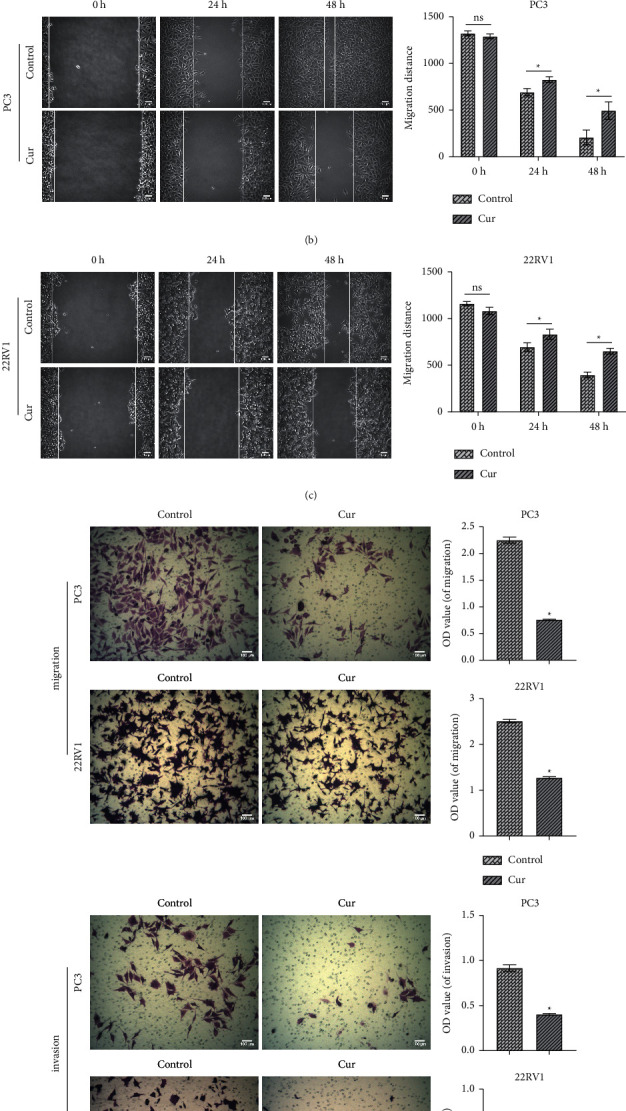
Effects of Cur treatment on biological processes of PC3 and 22RV1 cells. (a) The proliferation of RWPE-1 and PCa cells was detected by cell counting kit-8 (CCK8) assay. (b) and (c) The migration of PC3 and 22RV1 cells was tested by wound healing assay. (d) Transwell was utilized to measure the invasion of PC3 and 22RV1 cells (×100, scale bar = 100 *μ*m). ^*∗*^*P* < 0.05 vs. control group. ^#^*P* < 0.05 vs. 25 *μ*g/mL Cur. ^&^*P* < 0.05 vs. 50 *μ*g/mL Cur.

To determine the cytotoxicity of Cur and the drug sensitivity of PCa cells, Cur (0, 25, 50, and 100 *μ*g/ml) was used to treat RWPE-1 and PCa cells for 48 h. CCK8 results indicated that Cur had no significant effect on the proliferation of RWPE-1 cells. Compared with DU145 and LNCaP cells, PC3 and 22RV1 cells were the most sensitive to Cur (Figure 1(a)). In the 50 *μ*g/ml group, the comparison between different cell types was statistically significant. Therefore, PC3 and 22RV1 cells and 50 *μ*g/ml Cur were selected for subsequent experiments. As shown in Figures 1(b) and 1(c), after 24 h and 48 h, the migration ability of PC3 and 22RV1 cells was significantly inhibited by cur. Subsequently, we evaluated the invasion of PC3 and 22RV1 cells using the transwell assay. Compared with the control group, the invasion of PC3 and 22RV1 cells in the Cur group was notably decreased (Figure 1(d)).

### 3.2. Cur Upregulated the Expression of miR-125a

**Figure 2:**
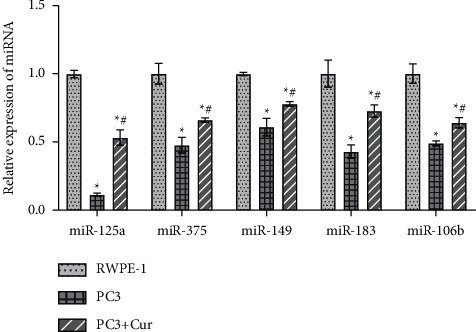
Cur upregulated the expression of miR-125a. qRT-PCR was performed to verify the effect of Cur on DE-miRNAs expression. ^*∗*^*P* < 0.05 vs. control group. ^#^*P* < 0.05 vs. Cur group.

By analyzing the GSE46738 and GSE45604 datasets, we found that 28 miRNAs were differentially expressed in the two datasets (Figures S1A–S1C). The GSE46738 dataset indicated that miR-125a was most downregulated in tumor tissues (logFC = −4.276611, Figure S1D). Gene Ontology (GO) and the Kyoto Encyclopedia of Genes and Genomes (KEGG) analysis found that the top three genes ontology-biological process (GO-BP) pathways of these DE-miRNAs included “leukocyte differentiation,” “negative regulation of reactive oxygen species metabolic process,” and “regulation of reactive oxygen species metabolic process.” The top three genes ontology-cellular component (GO-CC) pathways included “mitochondrial outer membrane,” “organelle outer membrane,” and “outer membrane.” The top three genes ontology‐molecular function (GO-MF) pathways included “phosphatase binding,” “protein phosphatase binding,” and “protein heterodimerization activity” (Figure S1E). In addition, the top three KEGG pathways included “MicroRNAs in cancer,” “Proteoglycans in cancer,” and “Human cytomegalovirus infection” (Figure S1F). Next, we examined the expression of these DE-miRNAs after Cur treatment in PC3 cells. qRT-PCR results showed that, compared with WPMY-1 cells, miR-125a, miR-375, miR-149, miR-183, and miR-106b were all downregulated in PC3 cells. After Cur intervention, miR-125a, miR-375, miR-149, miR-183, and miR-106b were all upregulated in PC3 cells, among which miR-125a was the most significantly upregulated (Figure 2).

### 3.3. miR-125a Targeted STAT3

**Figure 3:**
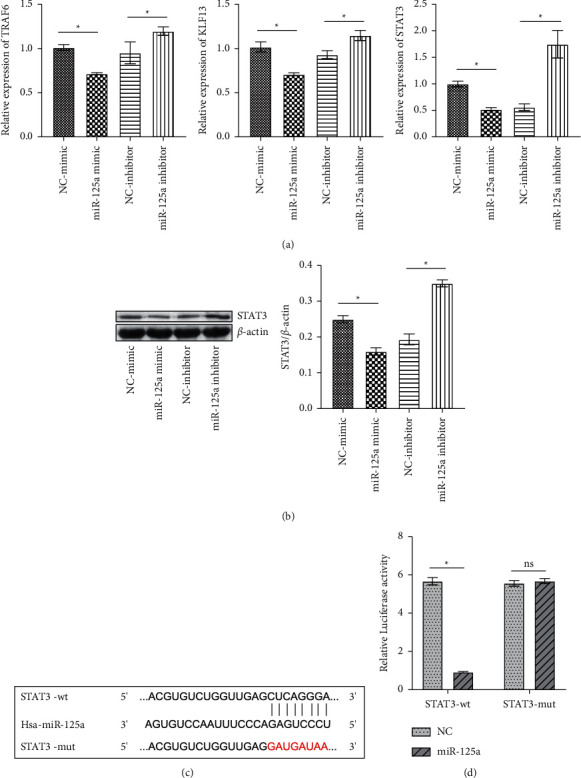
miR-125a targeted STAT3. (a) qRT-PCR was performed to verify the regulatory of miR-125a on TRAF6, KLF13, and STAT3. (b) WB was used to verify the regulatory of miR-125a on STAT3. (c) Bioinformatics analysis predicted the binding sites of miR-125a and STAT3. (d) Dual-luciferase reporter assay verified the binding sites of miR-125a and STAT3. ^*∗*^*P* < 0.05. ns: no significance.

The downstream target genes of miR-125a were predicted based on five databases. As shown in Figure S2, the three intersecting genes were found to be the signal transducer and activator of transcription 3 (STAT3), tumor-necrosis factor (TNF) receptor-associated factor 6 (TRAF6), and Kruppel-like factor 13 (KLF13). Then we downregulated and upregulated the expression of miR-125a-5p. We found that when the expression of miR-125a was overexpressed, the expression of STAT3 in mRNA level was significantly downregulated. When the expression of miR-125a was suppressed, the expression of STAT3 in mRNA level was significantly upregulated (Figure 3(a)). However, the expression changes of TRAF6 and KLF13 were not as significant as STAT3. Next, WB further verified the protein expression of STAT3. The results were consistent with qRT-PCR results (Figure 3(b)). Therefore, we selected STAT3 for follow-up studies. Bioinformatics analysis showed the binding sites between has-miR-125a-5p and STAT3 (Figure 3(c)). Dual-luciferase reporter assay revealed that miR-125a-5p targeted STAT3 (Figure 3(d)). Together, these findings indicated that miR-125a negatively regulated STAT3 expression.

### 3.4. Cur Inhibited the Activation of STAT3 Pathway

**Figure 4:**
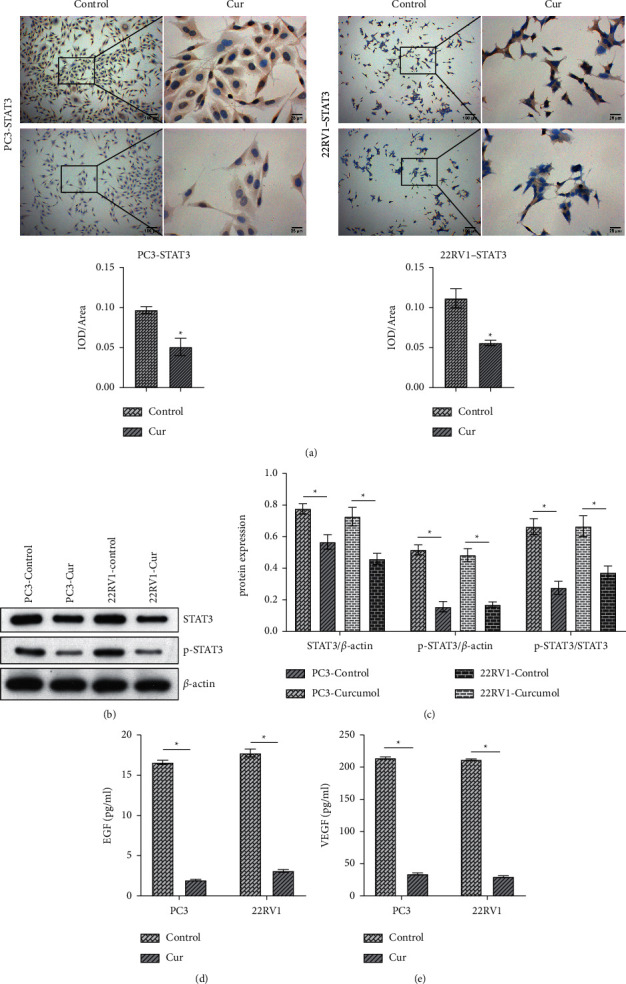
Cur inhibited the activation of STAT3 pathway. (a) ICC staining was used to test the expression of STAT3 in PC3 and 22RV1 cells. (b) and (c) WB was performed to verify the activation of the STAT3 pathway in PC3 and 22RV1 cells. (d) and (e) The levels of EGF and VEGF in PC3 and 22RV1 cells were measured by ELISA. ^*∗*^*P* < 0.05 vs. control group.

Through ICC experiments, we found that STAT3 expression in PC3 and 22RV1 cells was notably decreased in the Cur group compared with the control group (Figure 4(a)). WB analysis showed that Cur inhibited STAT3 phosphorylation in PC3 and 22RV1 cells (Figures 4(b) and 4(c)). Then STAT3 pathway downstream proteins (EGF and VEGF) were quantified by ELISA. As shown in Figures 4(d) and 4(e), the levels of EGF and VEGF were observably reduced in the Cur group compared to the control group. These results showed that Cur inhibited the activation of STAT3 pathway.

### 3.5. miR-125a Mediated the Regulation of Cur on STAT3

**Figure 5:**
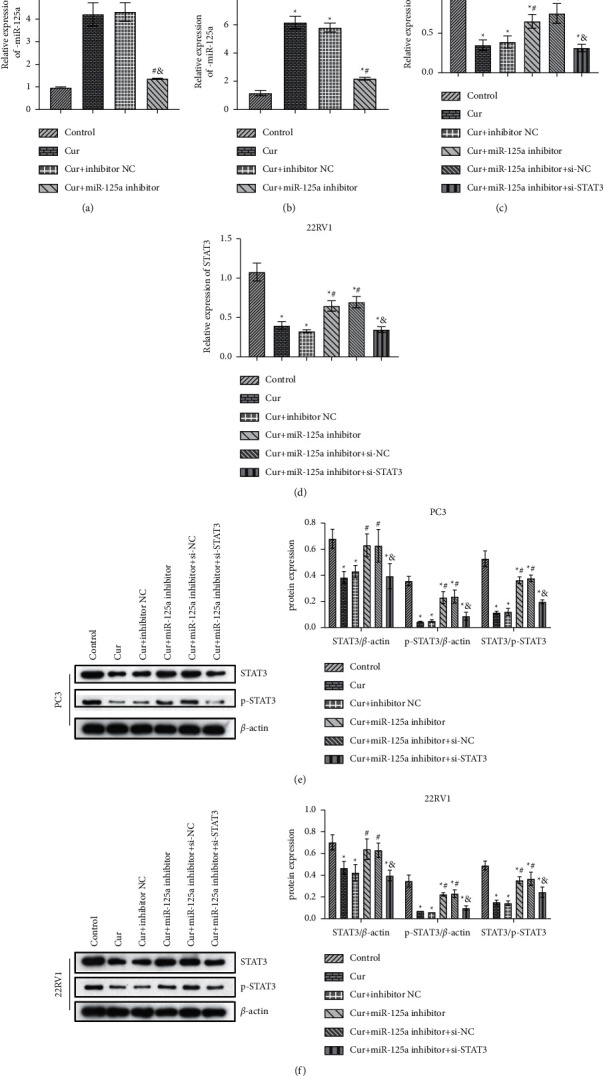
miR-125a mediated the regulation of Cur on STAT3. (a) and (b) qRT-PCR was used to detect the expression levels of miR-125a. (c) and (d) qRT-PCR was used to detect the expression levels of STAT3. (e) and (f) The expression of STAT3 and p-STAT3 was verified by WB. ^*∗*^*P* < 0.05 vs. control group. ^#^*P* < 0.05 vs. Cur. ^&^*P* < 0.05 vs. Cur + miR-125a inhibitor group.

Further, we explored whether Cur could regulate miR-125a to mediate the activation of STAT3 pathway. We first found that miR-125a inhibitor inhibited the upregulation of miR-125a in PC3 and 22RV1 cells by transfection of miR-125a inhibitor into PC3 and 22RV1 cells (Figures 5(a) and 5(b)). Furthermore, miR-125a inhibitor weakened the STAT3 inhibition of Cur in PC3 and 22RV1 cells, while si-STAT3 reversed this effect (Figures 5(c) and 5(d)). WB analysis showed that miR-125a inhibitor partially offset the inhibitory effect of Cur on STAT3 activation, and phosphorylation of STAT3 was significantly inhibited (Figures 5(e) and 5(f)). Taken together, we found miR-125a mediated the regulation of Cur on STAT3.

### 3.6. Cur Regulated the Proliferation, Migration, and Invasion of PC3 and 22RV1 Cells via miR-125a/STAT3 Axis

**Figure 6:**
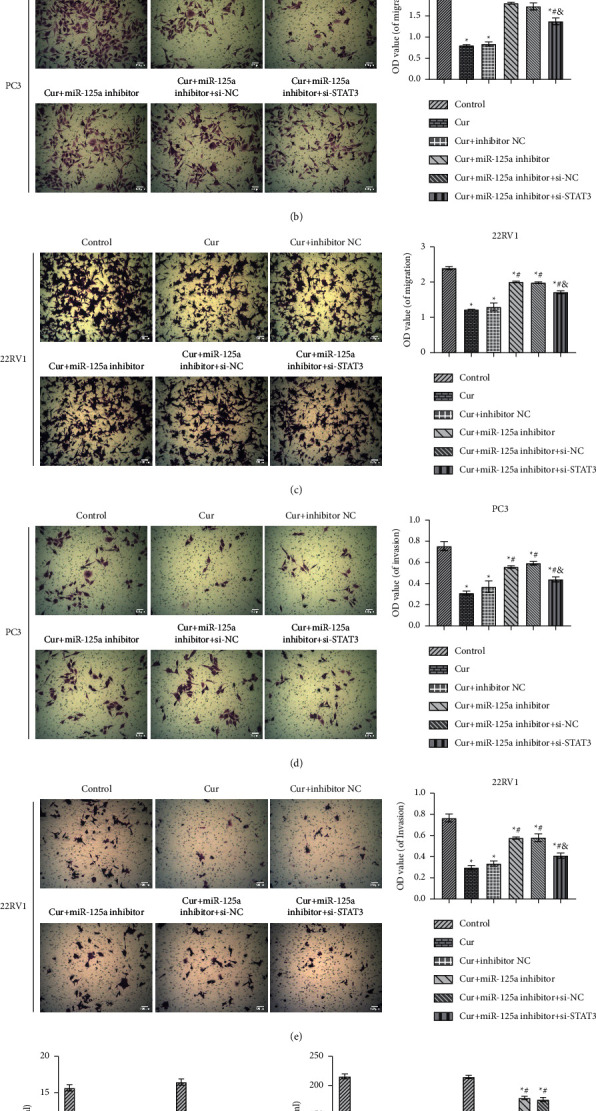
Cur regulated the proliferation, migration, and invasion of PC3 and 22RV1 cells via miR-125a/STAT3 axis. (a) The PC3 and 22RV1 cells activity was tested using CCK8. (b-c) Transwell assay was used to measure the migration of PC3 and 22RV1 cells (×100, scale bar = 100 *μ*m). (d-e) Transwell assay was used to measure the invasion of PC3 and 22RV1 cells (×100, scale bar = 100 *μ*m). (f) and (g) The levels of EGF and VEGF in PC3 and 22RV1 cells were quantified by ELISA. ^*∗*^*P* < 0.05 vs. control group. ^#^*P* < 0.05 vs. Cur. ^&^*P* < 0.05 vs. Cur + miR-125a inhibitor group.

We then functionally verified the regulatory approach of Cur in PCa *in vitro*. As shown in Figure 6(a), the cell activity was obviously restored in the Cur + miR-125a inhibitor group compared to the Cur group. Compared with the Cur + miR-125a inhibitor group, the cell activity was reduced in the Cur + miR-125a inhibitor + si-STAT3 group (Figure 6(a)). Analogously, PC3 and 22RV1 cells showed enhanced migration and invasion abilities in the Cur + miR-125a inhibitor group. In contrast, it was remarkably weakened in Cur + miR-125a inhibitor + si-STAT3 group (Figures 6(b)–6(e)). Although the levels of EGF and VEGF in PC3 and 22RV1 cells were reduced by Cur, they were partially recovered in cur + miR-125a inhibitor group. The subsequent transfection of si-STAT3 still reduced their levels (Figures 6(f) and 6(g)). These results revealed that Cur regulated the proliferation, migration, and invasion of PC3 and 22RV1 cells via miR-125a/STAT3 axis.

### 3.7. Cur Prohibited the Development of PCa by Regulating miR-125a/STAT3 Axis *In Vivo*

**Figure 7:**
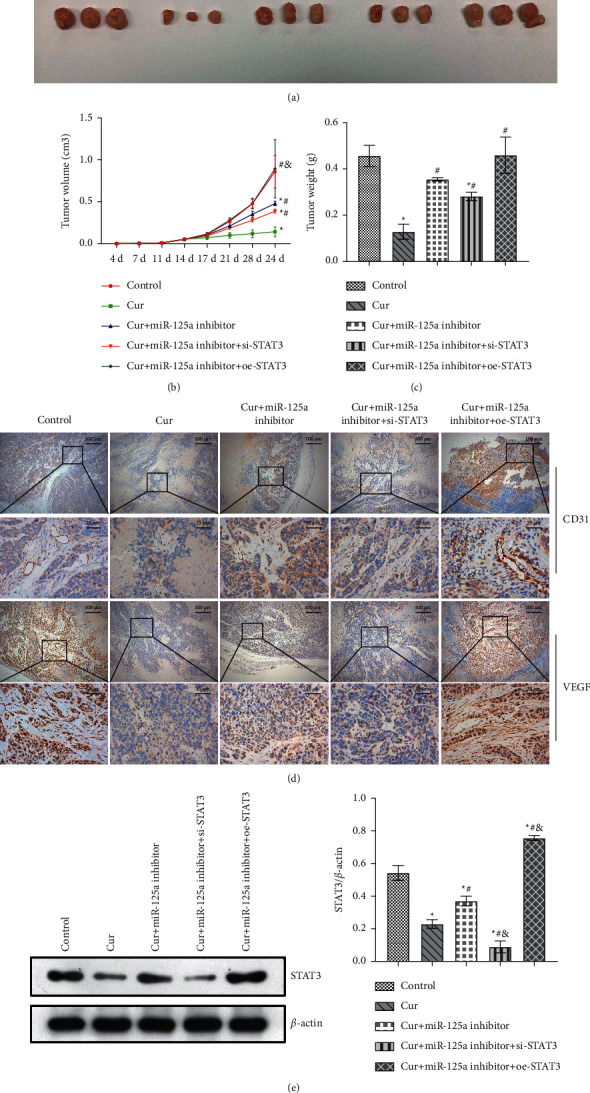
Cur prohibited the development of PCa by regulating miR-125a/STAT3 axis *in vivo*. (a) Tumor representative image. (b) Tumor volume. (c) Tumor weight. (d) IHC staining was used to evaluate the pathological status of CD31 and VEGF. (e) The expression of STAT3 in tumor was detected by WB. ^*∗*^*P* < 0.05 vs. control group. ^#^*P* < 0.05 vs. Cur. ^&^*P* < 0.05 vs. Cur + miR-125a inhibitor group.

Finally, we investigated the regulation mechanism of Cur on PCa development *in vivo*. We found that tumor growth rate and size were significantly inhibited in PCa mice treated with Cur. When miR-125a was repressed, the inhibitory effect of Cur on tumor was significantly weakened. Compared with the Cur + miR-125a group, tumor growth was partially reversed in the Cur + miR-125a inhibitor + si-STAT3 group. The tumor growth rate and size in the Cur + miR-125a inhibitor + oe-STAT3 group were further enhanced (Figures 7(a)–7(c)). CD31 is primarily used to demonstrate the presence of endothelial tissue and is used to assess tumor angiogenesis, which may indicate the extent of a rapidly growing tumor. IHC analysis indicated that CD31 and VEGF were markedly inhibited after Cur administration. After adding miR-125a inhibitor, the suppression of Cur on CD31 and VEGF was weakened. Compared with the Cur + miR-125a inhibitor group, the expression of CD31 and VEGF was inhibited or enhanced in Cur + miR-125a inhibitor + si-STAT3 or Cur + miR-125a inhibitor + oe-STAT3 group (Figure 7(d)). Moreover, STAT3 expression was significantly decreased after Cur administration, but miR-125a inhibitor partially reversed the inhibition of Cur (Figure 7(e)). These findings showed that Cur prohibited the development of PCa by regulating miR-125a/STAT3 axis *in vivo*.

## 4. Discussion

Cur was proved to be a plant compound with significant antitumor activity [23], which plays a crucial part in inhibiting cancer cell proliferation [24], apoptosis [25], metastasis [26], and epithelial mesenchymal transformation [27]. In this study, we explored the potential inhibitory mechanism of Cur on the occurrence and development of PCa. Furthermore, the putative targets and biological mechanisms of Cur regression in PCa were also determined *in vivo* and *in vitro*.

As far as we know, Cur has been shown to be therapeutic in many types of cancer. In our study, we also found that Cur memorably restrained the proliferation, migration, and invasion of PCa cells. There is increasing evidence that the antitumor effect of Cur may be mediated by miRNAs. In triple-negative breast cancer, Cur enhanced doxorubicin sensitivity by miR-181B-2-3p/ABCC3 axis to inhibit tumor growth [28]. In colorectal cancer, Cur inhibited tumor development by targeting miR-21 and modulating PTEN/PI3K/Akt pathways [29]. According to bioinformatics analysis, miR-125a was the most downregulated miRNAs in PCa. After Cur intervention, the miR-125a expression was observably upregulated. These findings imply that Cur may act on PCa through miR-125a.

Our previous article showed that Cur inhibited the malignant progression of prostate cancer and regulates the PDK1/AKT/mTOR pathway by targeting miR-9 [15]. Here, we conducted the study of miR-9 based on Ning N et al.'s report that Cur could regulate the expression of miR-9 [8]. Based on the research of Ning N et al., we previously verified the mechanism of Cur and miR-9 in PCa. However, in this study, we screened out miR-125a and the downstream target gene STAT3 through bioinformatics prediction and experimental verification. Then the subsequent verification of Cur and miR-125a/STAT3 axis was carried out. As a natural anticancer compound, Cur can regulate a variety of cellular mechanisms and inhibit or induce the production of multitudinous cytokines, growth factors, and enzymes, including STAT3, EGF, NF-*κ*B, cyclooxygenase II (COX-2), I*κ*B kinase *β* (I*κ*K*β*), protein kinase D1 (PKD1), mitogen-activated protein kinase (MAPK), and tumor-necrosis factor-*α* (TNF-*α*) [30, 31]. Through bioinformatics analysis and experimental verification, we found that miR-125a could negatively regulate STAT3. This aroused our great interest to explore the association between Cur and miR-125a/STAT3 axis. STAT3 is a nuclear protein associated with cell proliferation, which is capable of serving as an independent predictor of PCa metastasis and specific etiological mortality [32]. STAT3 is regulated by various posttranslational modifications [33]. We also found that miR-125a inhibitor partially offset the inhibitory effect of Cur on STAT3 activation, and phosphorylation of STAT3 was significantly inhibited. Therefore, we conjectured that Cur might affect the activation of the STAT3 pathway via regulating miR-125a.

Our results exhibited that the knockdown of miR-125a counteracted the inhibitory effect of Cur on cell proliferation, migration, and invasion, and further knockdown of STAT3 partially restored these effects. It demonstrated that Cur refrained from the activation of the STAT3 pathway by upregulating miR-125a. We also found that Cur effectively inhibited the growth PCa *in vivo*. These findings indicated that the resistance to PCa of Cur was mediated by the upregulation of miR-125a and inhibition of STAT3 phosphorylation.

## 5. Conclusion

Cur suppressed the development of PCa *in vivo* and *in vitro*. Moreover, Cur refrained from the proliferation, invasion, and migration of PCa cells through miR-125a/STAT3 axis. This may provide a new method and potential agent for the treatment of PCa.

## Figures and Tables

**Table 1:** Primer sequences.

Gene	Sequence (5′⟶3′)
U6	F CTCGCTTCGGCAGCACA R AACGCTTCACGAATTTGCGT
miR-125a	F TGCCAGTCTCTAGGTCCCTG R GCTCCCAAGAACCTCACCTG
miR-375	F CCGCGACGAGCCCCT R CCTCACGCGAGCCGAAC
miR-149	F CTGGCTCCGTGTCTTCACTC R CAGCTGCCCCAGCACAG
miR-183	F CGCAGAGTGTGACTCCTGTT R TGGCCCTTCGGTAATTCACT
miR-106b	F GGGGCTAAAGTGCTGACAGT R GGAGCAGCAAGTACCCACAG
STAT3	F CTCTTACTTCTCCAGCAACACT R ATACATGCTACCTAAGGCCAT
*β*-actin	F ACCCTGAAGTACCCCATCGAG R AGCACAGCCTGGATAGCAAC

## Data Availability

All datasets generated for this study are included within the article.

## Supplementary Materials

Supplementary Materials Figure S1. Differentially expressed microRNAs based on GSE46738 and GSE45604 datasets. (A) and (B) Volcano plots represent the up- and downregulated differentially expressed microRNAs (D-E-miRNAs) in GSE45604 and GSE46738, respectively. (C) Venn diagram showing the common and unique DE-miRNAs of GSE45604 and GSE46738. (D) Expression pattern of miR-125a in the GSE46738 microarray dataset. (E) and (F) Gene Ontology (GO) and Kyoto Encyclopedia of Genes and Genomes (KEGG) analyses based on the potential target genes of DE miRNAs. Figure S2. The downstream target genes of miR-125a were predicted based on five databases. The intersecting genes were found to be the signal transducer and activator of transcription 3 (STAT3), tumor-necrosis factor (TNF) receptor-associated factor 6 (TRAF6), and Kruppel-like factor 13 (KLF13).
