# Supplementary figures and images for: Curcumol Inhibits the Development of Prostate Cancer by miR-125a/STAT3 Axis
Source: Evid Based Complement Alternat Med. 2022 Jul 30;2022:9317402. doi: 10.1155/2022/9317402 (PMC9356804; doi:10.1155/2022/9317402)

A

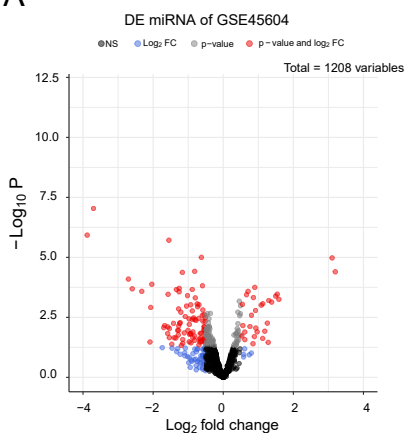

B

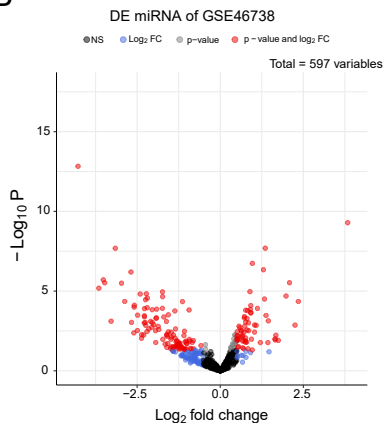

C

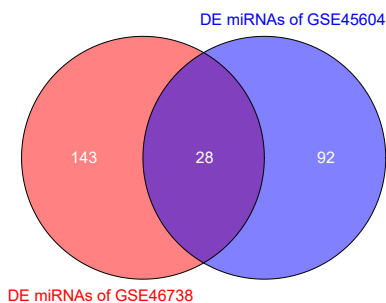

D

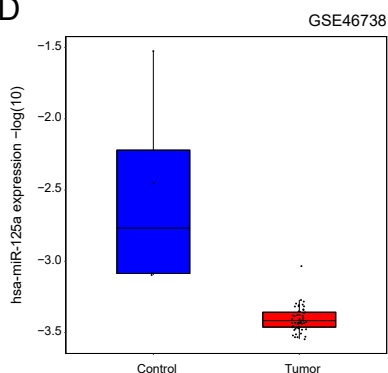

E

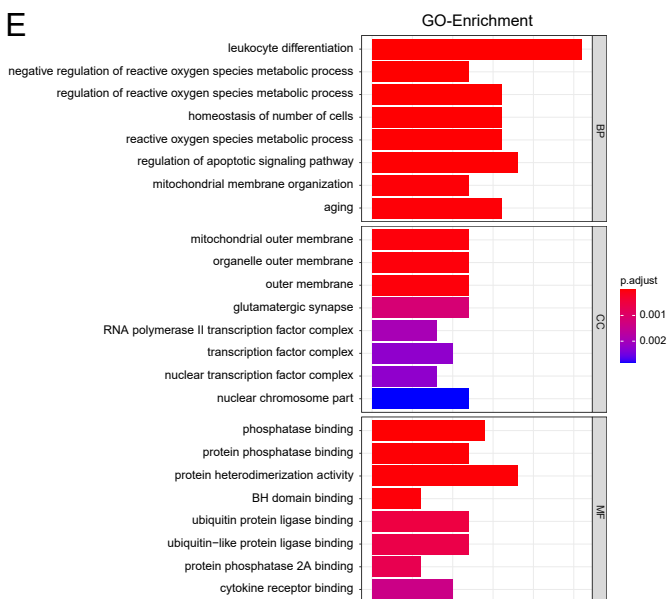

F

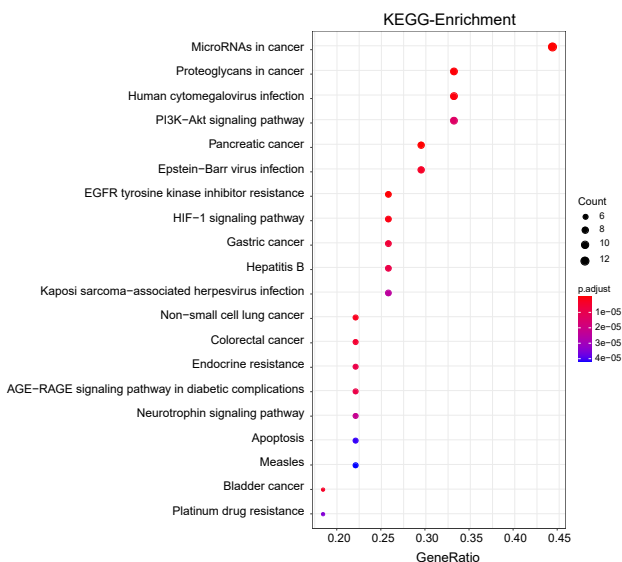

Supplement: Supplementary Materials — Figure S1. Differentially expressed microRNAs based on GSE46738 and GSE45604 datasets. (A) and (B) Volcano plots represent the up- and downregulated differentially expressed microRNAs (D-E-miRNAs) in GSE45604 and GSE46738, respectively. (C) Venn diagram showing the common and unique DE-miRNAs of GSE45604 and GSE46738. (D) Expression pattern of miR-125a in the GSE46738 microarray dataset. (E) and (F) Gene Ontology (GO) and Kyoto Encyclopedia of Genes and Genomes (KEGG) analyses based on the potential target genes of DE miRNAs. Figure S2. The downstream target genes of miR-125a were predicted based on five databases. The intersecting genes were found to be the signal transducer and activator of transcription 3 (STAT3), tumor-necrosis factor (TNF) receptor-associated factor 6 (TRAF6), and Kruppel-like factor 13 (KLF13). [file 9317402.f1.zip › Supplementary Materials/9317402.figure S1.pdf]

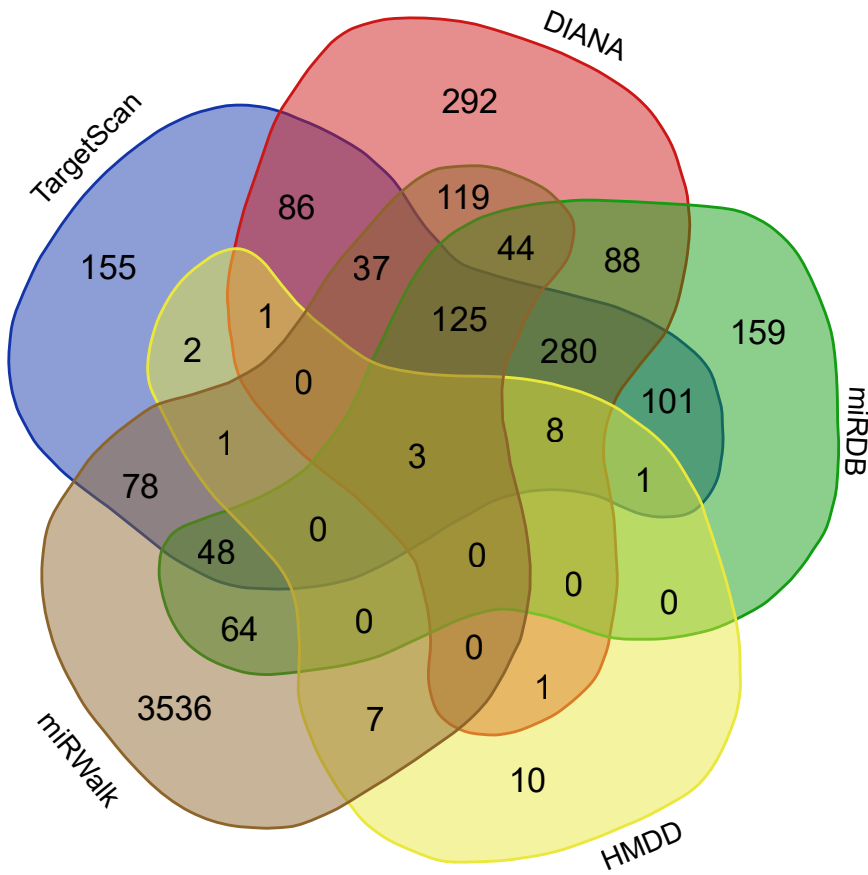

Supplement: Supplementary Materials — Figure S1. Differentially expressed microRNAs based on GSE46738 and GSE45604 datasets. (A) and (B) Volcano plots represent the up- and downregulated differentially expressed microRNAs (D-E-miRNAs) in GSE45604 and GSE46738, respectively. (C) Venn diagram showing the common and unique DE-miRNAs of GSE45604 and GSE46738. (D) Expression pattern of miR-125a in the GSE46738 microarray dataset. (E) and (F) Gene Ontology (GO) and Kyoto Encyclopedia of Genes and Genomes (KEGG) analyses based on the potential target genes of DE miRNAs. Figure S2. The downstream target genes of miR-125a were predicted based on five databases. The intersecting genes were found to be the signal transducer and activator of transcription 3 (STAT3), tumor-necrosis factor (TNF) receptor-associated factor 6 (TRAF6), and Kruppel-like factor 13 (KLF13). [file 9317402.f1.zip › Supplementary Materials/9317402.figure S2.pdf]
